# High-Efficiency Silicon Inverted Pyramid-Based Passivated Emitter and Rear Cells

**DOI:** 10.1186/s11671-020-03404-y

**Published:** 2020-08-28

**Authors:** Kun Gao, Ying Liu, Yuan Fan, Linxing Shi, Yufeng Zhuang, Yanfeng Cui, Shengzhao Yuan, Yimao Wan, Wenzhong Shen, Zengguang Huang

**Affiliations:** 1 School of Science, and School of Chemical Engineering, Jiangsu Ocean University, Lianyungang, 222005 Jiangsu Province People’s Republic of China; 2 Risen Solar Technology Co., Ltd., Changzhou, 213200 Jiangsu Province People’s Republic of China; 3 School of Physics and Astronomy, Key Laboratory of Artificial Structures and Quantum Control (Ministry of Education), Shanghai Jiao Tong University, Shanghai, 200240 People’s Republic of China

**Keywords:** Si solar cell, MACE, Inverted pyramids, PERC, High-efficiency

## Abstract

Surface texturing is one of the most important techniques for improving the performance of photovoltaic (PV) device. As an appealing front texture, inverted pyramid (IP) has attracted lots of research interests due to its superior antireflection effect and structural characteristics. In this paper, we prepare high-uniform silicon (Si) IPs structures on a commercial monocrystalline silicon wafer with a standard size of 156 × 156 mm^2^ employing the metal-assisted chemical etching (MACE) and alkali anisotropic etching technique. Combining the front IPs textures with the rear surface passivation of Al_2_O_3_/SiN_x_, we fabricate a novel Si IP-based passivated emitter and rear cell (PERC). Benefiting from the optical superiority of the optimized IPs and the improvement of electrical performance of the device, we achieve a high efficiency of 21.4% of the Si IP-based PERC, which is comparable with the average efficiency of the commercial PERC solar cells. The optimizing morphology of IP textures is the key to the improvement of the short circuit current *I*_sc_ from 9.51 A to 9.63 A; meanwhile, simultaneous stack SiO_2_/SiN_x_ passivation for the Si IP-based n^+^ emitter and stack Al_2_O_3_/SiN_x_ passivation for rear surface guarantees a high open-circuit voltage *V*_oc_ of 0.677 V. The achievement of this high-performance PV device demonstrates a competitive texturing technique and a promising prospect for the mass production of the Si IP-based PERC.

## Introduction

Improving efficiency is the eternal theme of the solar cell industry, which mainly focuses on two aspects: the optical performance and electrical performance. The front texturing technique is of importance for prompting the optical performance of the device. Inverted pyramid (IP) as an attractive light-trapping structure has attracted considerable attention due to its superior antireflection effect and structural characteristics [1–7]. To be specific, the incoming short-wavelength light in silicon (Si) IP undergoes triple or more bounces before being reflected away, possessing one or more bounces than that in traditional upright pyramids [7–9]. Meanwhile, this inverted pyramid-structured Si will avoid severe recombination losses faced by the nanostructured black Si [10–16] because of its big and open structural characteristic.

By employing the lithography inverted pyramid textures on the front surface and SiO_2_ passivation of the rear surface, Green’s group [17] has successfully fabricated a 25.0% efficient passivated emitter and rear local-diffused solar cell (PERL) with an area of 4 cm^2^. However, the lithography technique is not suitable for mass production because of its expense, low production-capacity, and incompatibility. Recently, many research interests turn to the metal-assisted chemical etching (MACE) large-area inverted pyramids since the MACE technique is simple, low-cost, large-area, and compatible with the current production line [14, 18–21]. For example, Jiang et al. [7] have reported inverted-pyramids nanostructure prepared by the MACE process followed by a post nanostructure rebuilding solution treatment and the conversion efficiency of IPs based multi-crystalline silicon (mc-Si) solar cells in large size of 156 × 156 mm^2^ wafers reached up to 18.62%. By utilizing Cu nanoparticles to catalyze chemical etching of Si, Yang et al. [8] have achieved 18.87% efficient IP-structured Si solar cells with a large area. Zhang et al. [9] have fabricated sc-Si solar cell with IP microstructure by modulated alkaline texturing combined with an optimized MACE method and have achieved a 20.19% efficient 1-μm-sized IP-textured device with a large area. So far, the performances of Si IP solar cell with a large area are not yet satisfied suffering from the large-area uniformity of IP morphology, the control of the IP feature size, and the passivation of the device. As a result, the front-optimized Si IP textures together with the rear passivation are expected to improve cell performance further.

In this paper, we successfully fabricated 21.4% efficiency Si IP-based passivated emitter and rear cells (PERC) with a standard solar wafer size of 156 × 156 mm^2^ by combining the front optimized MACE IP textures with the simultaneous stack SiO_2_/SiN_x_ passivation for the Si IP-based n^+^ emitter and stack Al_2_O_3_/SiN_x_ passivation for the rear surface. The key to high performance lies in the optical superiority of the IP textures and the reduced electrical losses by the simultaneous passivation of Si IP-based n^+^ emitter and rear surface. This novel Si IP-based PERC device structure and technique show a great potential in mass production of high-efficiency silicon-based solar cell.

## Methods

**Fig. 1:**
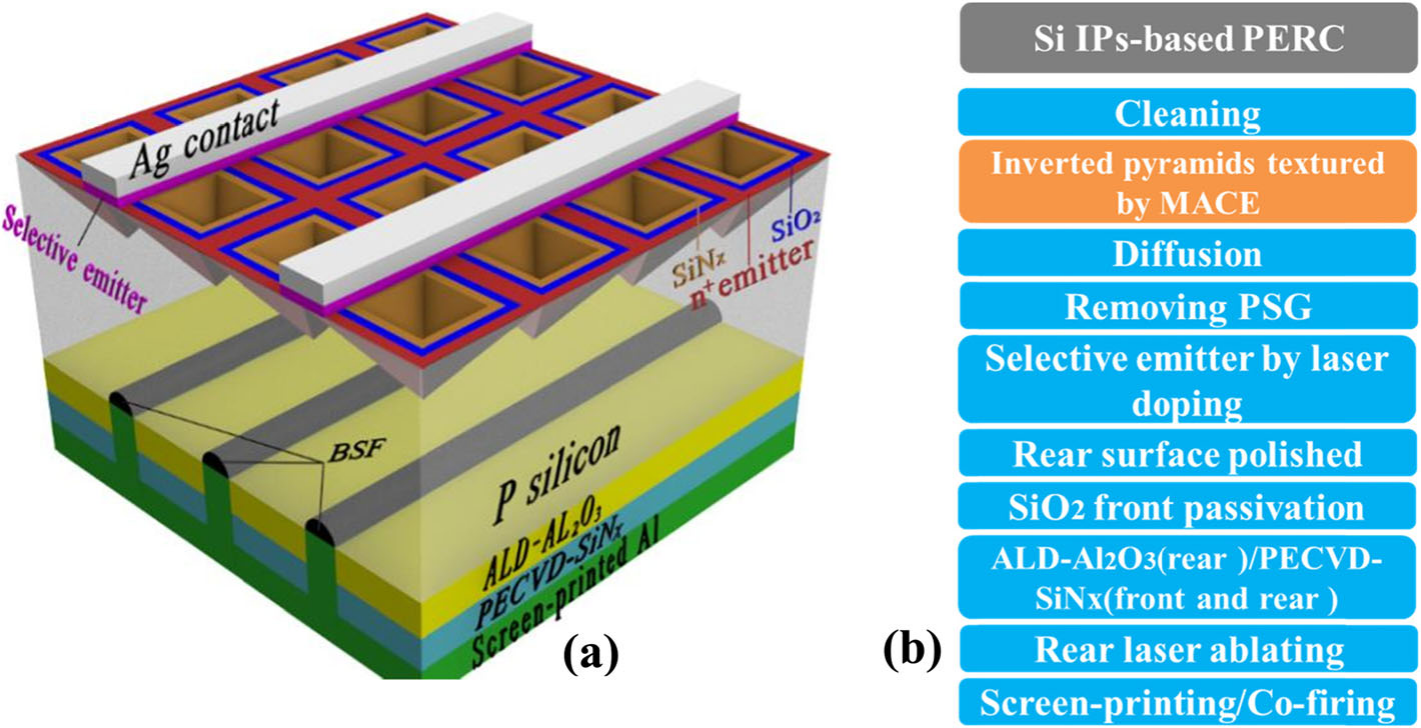
Design and process of the Si IP-based PERC. **a** Three-dimensional diagram of Si IP-based PERC. **b** Process flow of the Si IP-based PERC

The device structure of Si IP-based PERC is designed as follows: (i) The Si IP-based PERC n^+^ emitter is passivated by stack SiO_2_/SiN_x_ (PECVD) layers as shown in Fig. 1a. The Si IP structures have a good short-wavelength antireflection effect due to more opportunities of three or more bounces; meanwhile, the stack SiO_2_/SiN_x_ layer provides a further reduced reflectance and an excellent passivation effect for the Si IPs n^+^ emitter. (ii) The rear reflector is composed of stack Al_2_O_3_ (ALD)/SiN_x_ (PECVD) layers and screen-printed Al as shown in Fig. 1a. Stack dielectric layers are designed to optimize the optical properties of long-wavelength by increasing inner rear reflectance while maintaining a good electrical passivation effect, which is attributed to the field- effect passivation of the fixed negative charges in Al_2_O_3_ layer and the chemical passivation of hydrogen atoms in SiN_x_ film. In a word, both optical and electrical properties in this design are simultaneously considered to ensure a high performance of Si IP-based PERC.

Commercial 180-μm-thick 156 mm × 156 mm (100)-oriented crystalline silicon (c-Si), boron-doped (1–3 Ω·cm) p-type wafers were used as substrates. After the standard cleaning process, inverted pyramid textures were prepared on the surface of Si wafers as follows: (1) The cleaned Si wafers were immersed in the mixed solutions of AgNO_3_(0.0001 M)/HF (4 M)/H_2_O_2_ (1 M) for 300 s, resulting in porous Si. (2) Si wafers with porous Si were etched in an NH_4_OH:H_2_O_2_:H_2_O = 1:1:6 (volume) solutions for 200 s to remove the residual Ag nanoparticles. (3) The wafers with porous Si were modified in an HNO_3_:H_2_O:HF = 4:2:1 (volume) solution to prepare nanoholes. (4) Inverted-pyramids textures were fabricated on the surface of Si wafer by anisotropic etching of 60 °C-NaOH solutions for 30, 60, and 90 s, respectively.

POCl_3_ diffuses for 40 min at 800 °C in the quartz tube furnace and then n^+^ emitter forms on the front of the wafer (M5111-4WL/UM, CETC 48th Research Institute). The sheet resistance of Si IP-based n^+^ emitter is 105-110 Ω·sq^−1^. The selective emitter was fabricated on the front surface of the wafer by laser doping (DR-SE-DY70, DR Laser). After the rear surface polished, SiO_2_ passivation films were prepared by thermal oxidation on the front of silicon wafers. The Al_2_O_3_ passivation layers were deposited on the rear surface of wafer by ALD (PEALD-156, HUGUANG Scientific Instruments of Beijing) for ≈ 30 min at 150 °C. The PECVD-SiN_x_ layers were formed by the reaction of NH_4_/SiH_4_ (SC-TD-450C). Subsequently, the rear stack passivation layers of Si IP-based wafer were locally ablated by a 532-nm wavelength and 10-ps pulse length laser (DR-AL-Y60, DR Laser), in order to form the 50-μm width and 1-mm pitch local line openings. Finally, the Si IP-based PERC underwent the commercial screen-printing (PV1200, DEK) and co-firing process (CF-Series, Despatch), to form well Ohmic contacts and local BSFs.

The morphologies and structures of the samples were characterized with a JEOL JSM-6390LA scanning electron microscope. The lifetime of the minority carriers was measured by using a Sinton WCT-120. The absorption spectra were determined by FTIR (Tensor 27, BRUKER). The C-V curve is measured by an impedance analyzer (E4900A, KEYSIGHT). The photoluminescence and electroluminescence photos were taken by PL/EL imaging analysis system (LIS-R2, BTimaging). The reflectance spectra, as well as the IQEs and EQEs, were measured on the platform of quantum efficiency measurement (QEX10, PV Measurements). The electrical parameters of the solar cells were investigated by current-voltage (I–V) measurement under the illumination of AM1.5 (Crown Tech IVTest Station 2000). The cell efficiency was measured by using a BERGER Lichttechnik Single Cell Tester.

## Results and Discussion

**Fig. 2:**
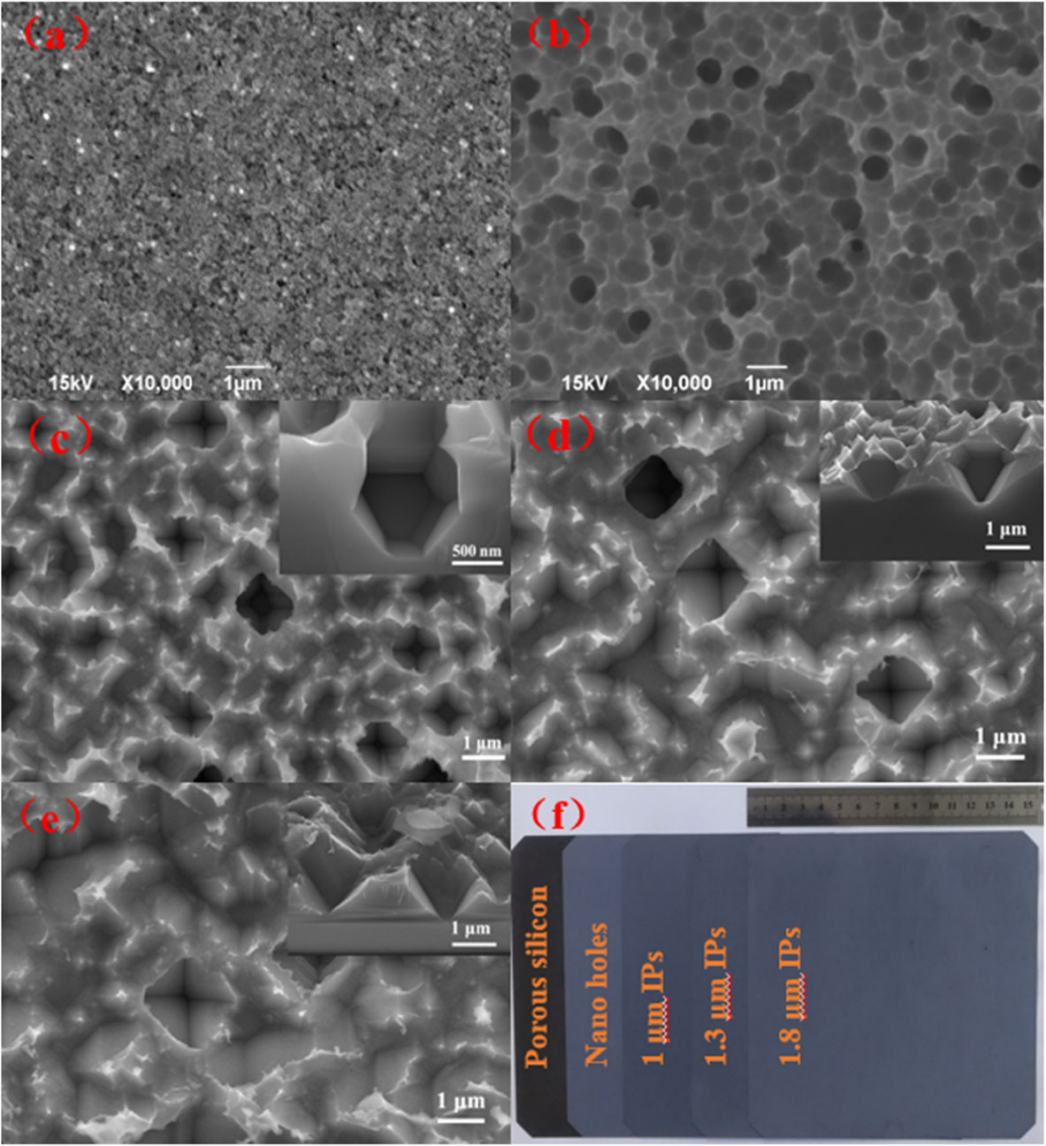
Morphology of the prepared Si inverted pyramid structures (Si IPs-strus). **a** SEM image of porous silicon obtained by MACE. **b** SEM image of nanoholes by the following modifications in the HF/HNO_3_ mixed solutions. **c**–**e** SEM images of inverted pyramids (cross section in inset) by the etching in aqueous NaOH solution at 60 °C for 30, 60, and 90 s, respectively. **f** Compared photos for different surface structures corresponding to **a**–**e**

Figure 2a–e shows the top-view SEM images of the different process steps for the silicon surface texturing. Figure 2a shows the 50–80 nm porous Si on the surface of Si wafer etched by MACE method in the mixed solutions of AgNO_3_/HF/H_2_O_2_. Subsequently, the porous Si is modified by the isotropic etching in the mixed aqueous solutions containing HF/HNO_3_ and turns to be nanohole structures with a diameter of 800 nm as shown in Fig. 2b. Finally, the micron inverted pyramids (IPs) with different sizes (Fig. 2c–e) are obtained by sodium hydroxide in aqueous solution at 60 °C for 30, 60, and 90 s, respectively. From Fig. 2c–e, we can see that after alkali treatment, the IPs structure sizes for three etching time of 30, 60, and 90 s are ~ 1, 1.3, and 1.8 μm, respectively, meaning an increasing size of IP with the increase of alkali treatment time. Also, we notice that the IPs tend to collapse and transit to be the upright pyramids with the increase of the etching time. As known, the inverted pyramids have the advantage of light trapping over upright ones because light will undergo extra one or two bounces in inverted pyramids than that in upright pyramids. Therefore, the structures with shorter etching time are suitable for the light-trapping textures of PV devices because of the advantage in the short-wavelength antireflection. Figure 2f is the compared photos for different surface structures corresponding to Fig. 2a–e.

**Fig. 3:**
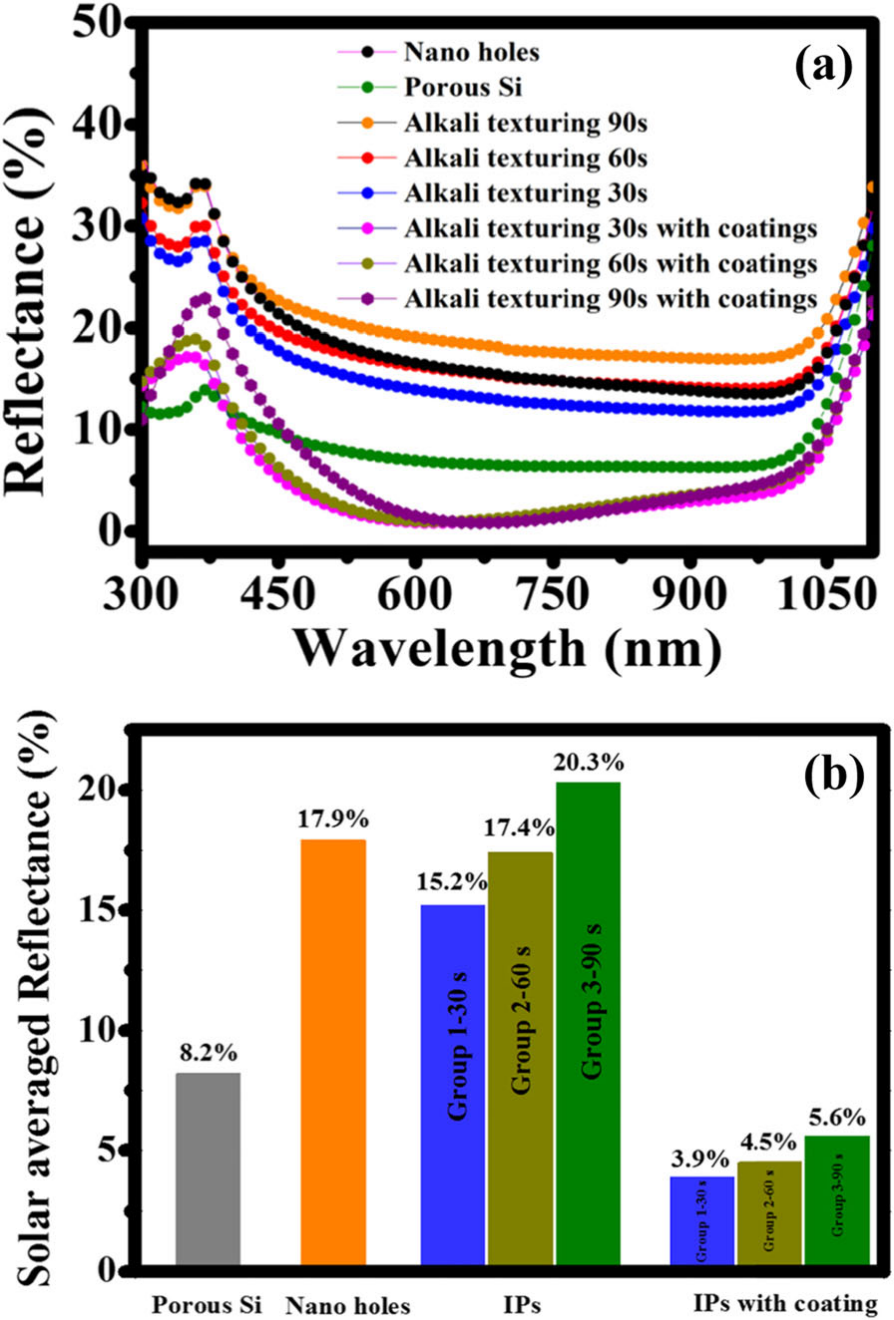
Optical properties of the prepared Si IP-strus. **a** The measured reflectance of different surface morphology and **b** the solar averaged reflectance *R*_ave_ over the 300–1100 nm wavelength range

Now we turn to the optical properties of Si IP-strus. From the reflectance over the whole wavelength range of 300–1100 nm (Fig. 3a), we observe that the porous Si has a low reflection because of the excellent light-trapping performance of nanostructures [22–24]. For nanohole structures, the reflectance in the whole wavelength range has an obvious increase, which is attributed to the decrease of density and increase of feature size of nanoholes. After NaOH treatment for 30 s, benefiting from 3–4 bounces between the (111) planes of the IP, the IPs structures display lower reflection over the 300–1100 nm wavelength range, especially in the short-wavelength range of 300–500 nm. With the alkali etching time increasing, the IPs become larger and tend to be the upright pyramids, resulting in an increasing reflectance. When all samples were covered with the same stack SiO_2_/SiN_x_ coating, the reflectance drop sharply by more than 10%, which is attributed to the combined reflectance from the optical interference of the stack SiO_2_/SiN_x_ thin films and the surface structures. In this case, the reflection spectra of samples from different processes are mainly different in the wavelength range of 300–600 nm, which is caused by the difference of feature size of IPs. In particular, Si IP-strus covered by the stack SiO_2_/SiN_x_ layers displays better short-wavelength antireflection ability than the others, indicating the excellent external quantum efficiencies (EQEs) in the short-wavelength range.

Furthermore, we calculate the average solar reflectivity *R*_ave_ (see Fig. 3b) over the wavelength range of 300–1100 nm and compare the reflectivity of Si IP-strus with other structures corresponding to different intermediate processes shown in Fig. 2a–c. *R*_ave_ can be calculated by the expression of

1 $$ R\mathrm{ave}=\frac{\int_{300\ \mathrm{nm}}^{1100\ \mathrm{nm}}\mathrm{R}\left(\uplambda \right)\ast \mathrm{S}\left(\uplambda \right)\ast \mathrm{d}\uplambda}{\int_{300\ \mathrm{nm}}^{1100\ \mathrm{nm}}\mathrm{S}\left(\uplambda \right)\ast \mathrm{d}\uplambda} $$

where *R*(*λ*) and *S*(*λ*) denote the measured reflectance and AM1.5 solar photon spectral distribution, respectively. As shown in Fig. 3b, the *R*_ave_s of porous Si, nanoholes, IPs, and IPs with SiO_2_/SiN_x_ coating are 8.22, 17.96, 15.18 (group 1—30 s)/17.35% (group 2—60 s)/20.3% (group 3—90 s), and 3.91% (group 1—30 s)/4.48% (group 2—60 s)/5.60% (group 3—90 s), respectively. The *R*_ave_s show that the IP-strus have a better antireflection ability than nanoholes and show a decreasing trend with the increase of feature size. When IP-Strus are coated by the stack SiO_2_/SiN_x_ layers, the lowest *R*_ave_ is 3.91%, revealing an ideal light-trapping structure for the PV device.

**Fig. 4:**
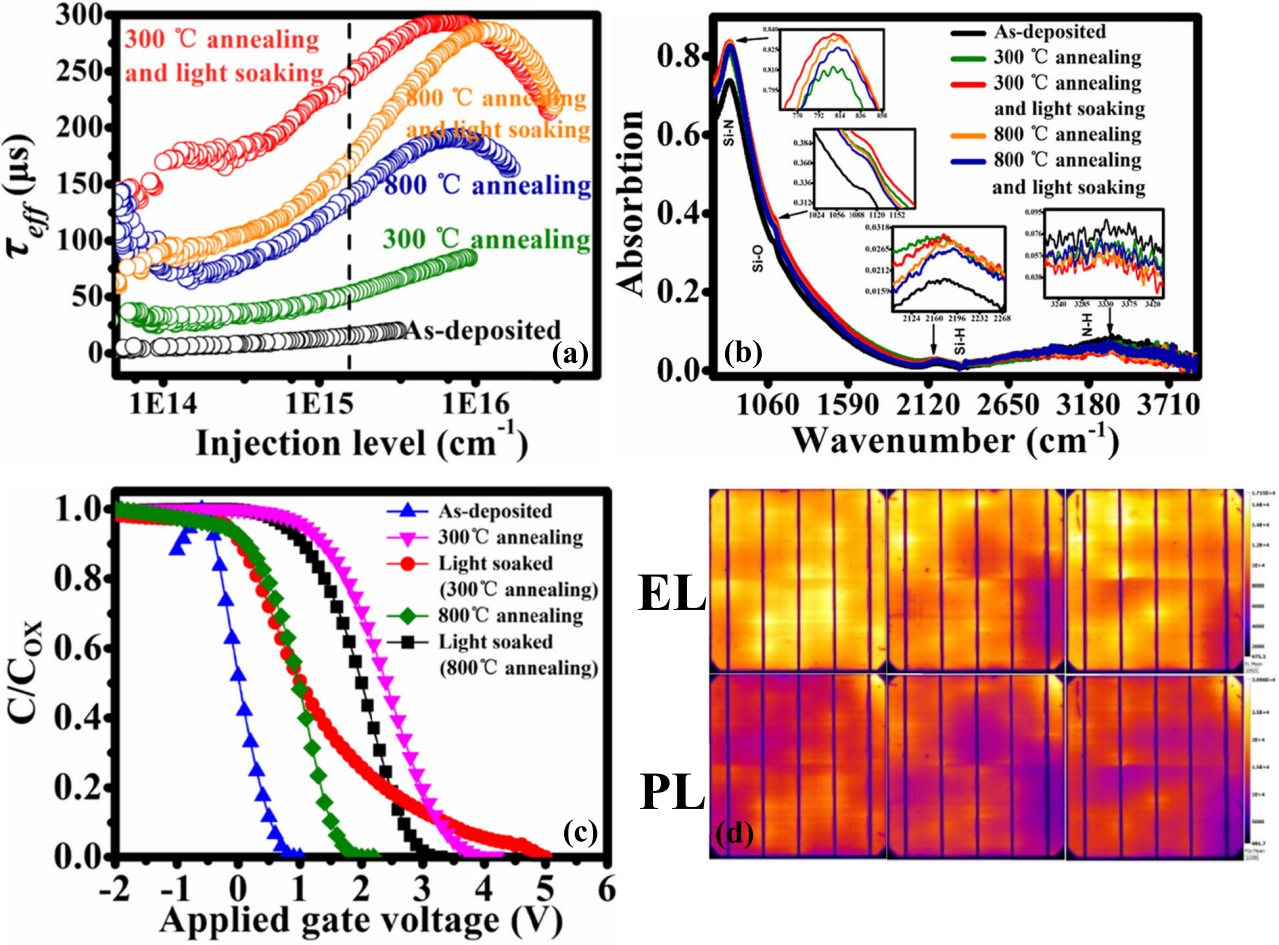
**a** *τ*_eff_ with respect to the injection level *Δn* at different annealing temperatures for Al_2_O_3_/SiN_x_ passivated wafers. The dashed line denotes one sun injection level. **b** The FTIR spectra of the samples. **c** *C–V* curves for the Au/Al_2_O_3_-SiN_x_/Si structure. **d** Photoluminescence and electroluminescence photos of devices

The stack SiO_2_ (~ 2 nm)/SiN_x_ (~ 75 nm) passivation for the Si IP-based n^+^ emitter is an effective way for achieving well electrical performance of IP-based PERC and their passivation effect [1] and mechanism have been systematically studied in our previous work [14]. To show the electrical superiority of the stack Al_2_O_3_/SiN_x_ passivation layers at the rear of our device, we investigate the influence of the different annealing and light-soaking conditions on the effective minority carrier lifetime (*τ*_eff_) with respect to the injection level (*Δn*), as shown in Fig. 4a. Notice that the polished Si wafers have the bulk minority carrier lifetime of ~ 350 μs, and the stack Al_2_O_3_/SiN_x_ layers are symmetrically deposited on both sides of polished Si wafers. The thickness of inner Al_2_O_3_ and the outer SiN_x_ layer is estimated as ~ 3 and ~ 125 nm, respectively. Two annealing conditions are performed in the air atmosphere: 300 °C and 800 °C for 15 min. Then the wafers are illuminated at 25 °C under the full-wave ranged halogen lamp with a power intensity of 50 mW cm^−2^ for 100 s. As can be seen from Fig. 4a, the 48 μs *τ*_eff_ (300 °C) and 126 μs *τ*_eff_ (800 °C) after annealing are much higher than the 22 μs *τ*_eff_ of the as-deposited Al_2_O_3_/SiN_x_ passivated samples at the injection level of 1.2 × 10^15^ cm^−3^.

Importantly, the effective minority lifetime of annealed samples after 100 s of illumination are 230 μs and 150 μs, respectively, much higher than 126 μs and 48 μs before illumination, demonstrating a very clear light-enhanced c-Si surface passivation of Al_2_O_3_/SiN_x_ layers. The charge trapping effect during light soaking [25–28] could be one of the main mechanisms for the light-enhanced c-Si surface passivation of Al_2_O_3_/SiN_x_ films. As Al_2_O_3_ films are reported to have a negative fixed charge density [29–32], some of the excess electrons generated by light were likely to be injected or tunneled into trap states in the inner Al_2_O_3_ film, resulting in an increased level of field-effect passivation. Interestingly, the light-enhanced passivation effect at 300 °C annealing is better than that at 800 °C, meaning that light-soaking at a lower temperature annealing is a more effective way to the application of PV device.

To study the effect of the annealing process on the surface modification, we compare the Fourier transform infrared spectroscopic (FTIR) absorption spectra of the annealed samples with that of the as-deposition sample. Figure 4b manifests that the Si–N, Si–O, Si–H, and N–H bonds correspond to the stretching absorption peaks at the wavenumbers of ~ 840, 1070, 2200, and 3340 cm^−1^, respectively. We see that the densities of both the Si–N and Si–O bonds show an obvious increase after annealing; meanwhile, the density of the Si–H bonds increases slightly. The increases of the Si–O and Si–H bond density implies the decrease of the dangling bonds at the interface of Si/SiO_2_, resulting in a better passivation effect [33]. Also, the annealing process promotes the density of Si–N bonds, indicating a more dense structure which can effectively prevent the out diffusion of H from entering into the environment instead of into Si bulk. However, for excessively high annealing temperature, the H in Si–H and N–H groups can escape from the bulk Si and the dielectric layers to the environment, which causes the decline of the passivation effect. The result of FTIR is consistent with that of the effective minority lifetime.

To further understand the difference of passivation mechanism between thermal annealing and light-soaking treatment, we analysis the density of fixed charges (*N*_*f*_) and the density of interface traps (*N*_it_) at the interface of Si and Al_2_O_3_ (ALD)/SiN_x_ (PECVD) stack layers by using capacitance–voltage (*C-V*) measurements from a rigorous metal–oxide– semiconductor (MOS) model.

*N*_*f*_ can be obtained from the following equation:

2 $$ {\mathrm{N}}_{\mathrm{f}}=\frac{{\mathrm{Q}}_{\mathrm{f}}}{\mathrm{S}\times \mathrm{e}}=\frac{{\mathrm{C}}_{\mathrm{OX}}\times \left({\mathrm{V}}_{\mathrm{MS}}-{\mathrm{V}}_{\mathrm{FB}}\right)}{\mathrm{S}\times \mathrm{e}} $$

where the following expression can calculate V_FB_

3 $$ {V}_{\mathrm{FB}}={V}_{\mathrm{MS}}-\frac{Q_f}{C_{\mathrm{OX}}} $$

Note that *S* is the area of metal electrode, *e* is electronic charge, *C*_OX_ is the capacitance of dielectric film layer, *V*_MS_ is the difference of the work function between the metal electrode and p-type Si, and *V*_FB_ is flat band voltage.

Using the Lehovec method [34], we can obtain *N*_it_ from the *C-V* curve:

4 $$ {\mathrm{N}}_{\mathrm{it}}=\frac{\left({\mathrm{C}}_{\mathrm{OX}}-{\mathrm{C}}_{\mathrm{FB}}\right){\mathrm{C}}_{\mathrm{FB}}}{3{\left(\updelta \mathrm{C}/\updelta \mathrm{V}\right)}_{\mathrm{FB}}\mathrm{ekTS}}-\frac{{\mathrm{C}}_{\mathrm{OX}}^2}{\left({\mathrm{C}}_{\mathrm{OX}}-{\mathrm{C}}_{\mathrm{FB}}\right)\mathrm{S}{\mathrm{e}}^2} $$

where (*δC*/*δV*)_FB_ is the slope near-flat band and is taken as the absolute value. *C*_FB_, *e*, and *k* are capacitance of MOS structure in a flat band, electronic charge, and Boltzmann constant, respectively.

It can be seen from Fig. 4c that the measured *C-V* curve of the Al_2_O_3_/SiN_x_ stack layers shows obvious accumulation region, depletion region, and inversion region. According to the *C-V* curves and Eq. (2–4), we obtain the interface properties of the prepared MOS structures, as shown in Table 1.

**Table 1:** *N*_*f*_ and *N*_it_ at the interface between Al_2_O_3_/SiN_x_ stack layers and Si

Sample	*N*_*f*_/cm^−2^	*N*_it_/cm^−2^ eV^−1^
As-deposited	− 4.3 × 10^11^	3.89 × 10^12^
300 °C annealing	− 2.26 × 10^12^	8.59 × 10^11^
300 °C annealing and light soaking	− 2.87 × 10^12^	8.68 × 10^11^
800 °C annealing	− 1.04 × 10^12^	4.32 × 10^11^
800 °C annealing and light soaking	− 1.65 × 10^12^	4.65 × 10^11^

The fixed negative charge densities show a significant increase by an order of magnitude after thermal annealing meanwhile the interfacial states densities significantly decrease, indicating that annealing enhanced the chemical passivation and field-effect passivation of dielectric films. By further light-soaking treatment, the densities of interfacial states keep the same level, while the densities of fixed negative charges increase further. As mentioned above, some of the excess electrons generated by light were likely to be injected or tunneled into trap states in the inner Al_2_O_3_ film, which means that light soaking can enhance the field-effect passivation of the dielectric film. Although the value of *N*_it_ is high, the sample by 300 °C annealing and 100 s light-soaking has the highest *τ*_eff_ of 230 μs due to the highest *N*_*f*_ of − 2.87 × 10^12^ cm^−2^, meaning that field-effect passivation has an advantage over chemical passivation in this case.

Figure 4d shows the photoluminescence and electroluminescence photos of 1, 1.3, and 1.8 μm IP solar cells with the same passivation process. The brightness of the three groups of photos for both photoluminescence and electroluminescence keeps basically the same level, meaning that the three groups of solar cell devices perform equally well in the passivation of defects. That is to say, the passivation process determines the electrical performance of the solar cell instead of the feature size of IPs, which will be confirmed by the following output parameters of the fabricated solar cells.

Based on the excellent optical and electrical performance of the simultaneous SiO_2_/SiN_x_ stack layers passivated front Si IP-based n^+^ emitter and Al_2_O_3_/SiN_x_ stack layers passivated rear reflector, we fabricated the Si IPs-based PERC.

**Fig. 5:**
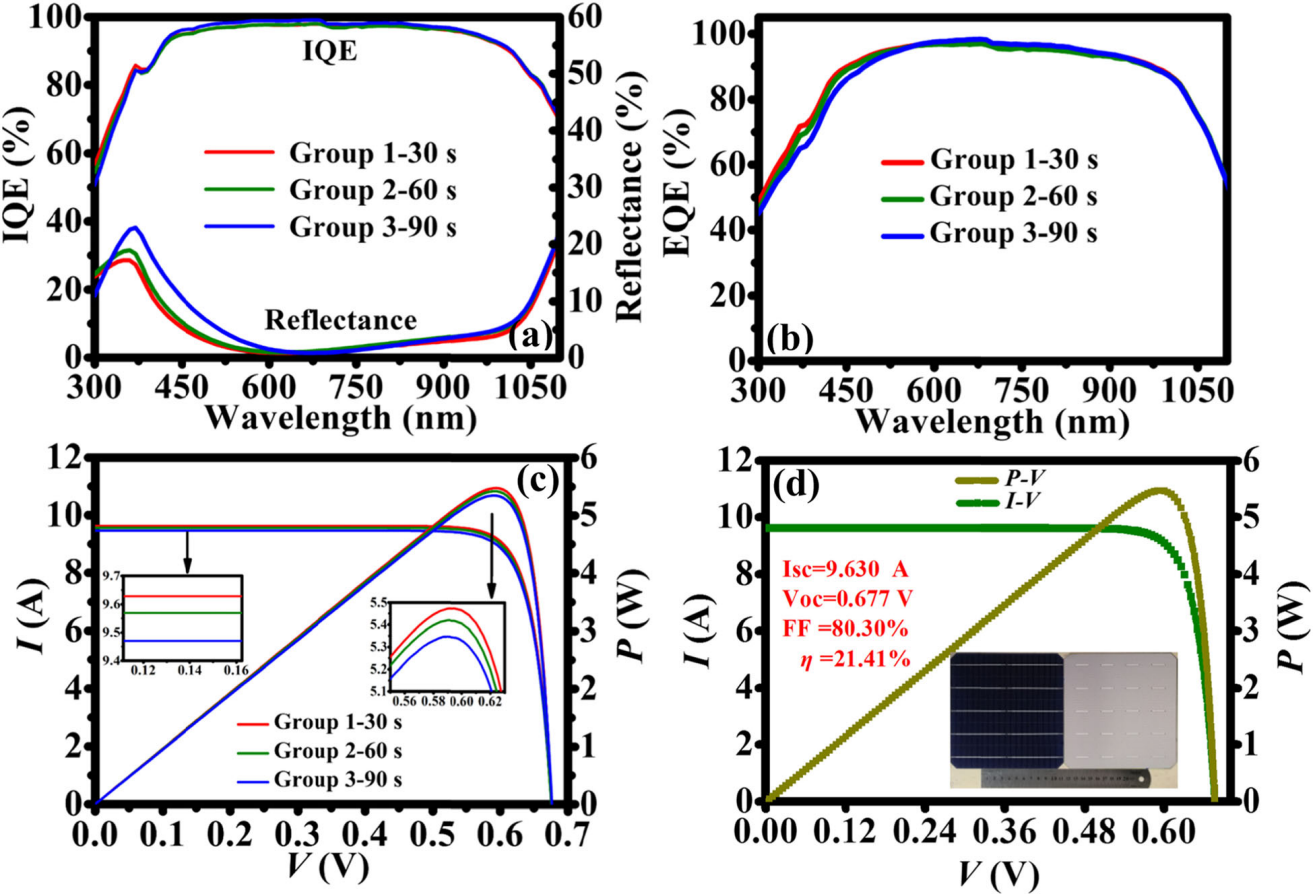
High-performance Si IP-based PERC. **a** The IQE and reflectance of the Si IP-based PREC with different alkaline etching times. **b** The EQE of the Si IP-based PERC with different alkaline etching time. **c** The *I–V* and *P-V* curve of the Si IP-based PERC with different alkaline etching time. **d** *I–V* and *P-V* curve of the champion device

Figure 5a shows the internal quantum efficiencies (IQEs) and front surface reflections of the fabricated Si IP-based PERCs. We can observe that 30-s alkali-etching IP-based device (group 1—30 s) shows the lowest reflectance in the short wavelength of 300–600 nm due to its smaller feature size of IPs. Importantly, group 1—30 s has the highest IQEs in this wavelength range, and thus yields the highest external quantum efficiencies (EQEs) as shown in Fig. 5b. Also, the fabricated devices display almost the same EQEs in the long-wavelength range because of the same level of reflectance and IQEs in this range. Therefore, group 1—30 s with smaller feature size possesses better output performance than the other two groups, which is further confirmed by the *I-V* and *P-V* curves of devices (see Fig. 5c). Figure 5d shows the *η* of our champion device reached 21.41%, as well as the *V*_oc_ of 0.677 V, *I*_sc_ of 9.63 A, and *FF* of 80.30%. By our knowledge, it is the highest *η* among MACE-IP-based solar cells. The inset of Fig. 5d is a photograph of the front and rear surface of the champion device.

Furthermore, Table 2 shows the detailed parameters of the fabricated devices. Obviously, the average *I*_sc_ (9.63 A) of the group 30 s device is higher than that of the other two groups, which lies in its best anti-reflection ability of front surface as mentioned above. The difference of *I*_sc_s mainly determines the output performances of the devices. Besides, the higher *FF* and the lower series resistance *R*_*s*_ guarantees the higher *η* of group 30 s. It is worth to note that all the average *V*_oc_s of the Si IP-based PERCs are in the range of 674–676 mV, demonstrating that the same excellent passivation for the front and rear surface of all groups. Finally, benefiting from the gain of optical and electrical performance, we have successfully achieved the highest *η* of 21.4% of Si IP-based PERC solar cell.

**Table 2:** Detailed output parameters of Si IPs-based PERC

Group		*V*_oc_/mV	*I*_sc_/A	*R*_*s*_/Ω cm^2^	*FF*/%	*η*/%
1—30 s	Best	677	9.63	0.002429	80.30	21.4
	Average	676	9.62	0.002497	80.15	21.3
2—60 s	Best	676	9.60	0.002521	80.14	21.3
	Average	674	9.57	0.002499	80.01	21.1
3—90 s	Best	679	9.51	0.002576	80.26	21.2
	Average	676	9.49	0.002603	79.94	21.0

## Conclusions

In conclusion, we optimize the morphologies of the MACE Si IPs structures and fabricate the novel Si IPs-based PERC solar cell with a standard size of 156 × 156 mm^2^ by combining the stack SiO_2_/SiN_x_ layers coated IPs textures with the stack Al_2_O_3_/SiN_x_ passivation of the rear surface. The optical properties show that the solar averaged *R*_ave_ of IPs textures coated by the stack SiO_2_/SiN_x_ layers can be up to 3.91%, revealing IPs an ideal light-trapping structure for PV device. Also, the electrical analysis shows that the polished rear surface passivated by the stack Al_2_O_3_/SiN_x_ layers possess very high *τ*_eff_ of 230 μs due to the thermal and light-soaking treatment, demonstrating well light-enhanced c-Si surface passivation of Al_2_O_3_/SiN_x_ layers. FTIR measurements provide a further explanation for the high *τ*_eff_s of the rear surface passivated by the stack Al_2_O_3_/SiN_x_ layers. Importantly, a high fixed charge density *N*_*f*_ of − 2.87 × 10^12^ cm^−2^ is obtained by means of the C-V measurements, which reveals strong field-effect passivation of Al_2_O_3_/SiN_x_ layers. Finally, benefiting from the excellent optical and electrical performance at the front Si IP-based n^+^ emitter and rear reflector, we achieve the highest η of 21.4%, as well as *V*_oc_ of 0.677 V, *I*_sc_ of 9.63 A, and *FF* of 80.30%. The achievement of high-efficiency Si IP-based PERC provides IPs with an effective way to mass production of Si-based high-efficiency solar cells.

## Data Availability

The datasets supporting the conclusions of this article are included within the article.

## Abbreviations

PV: Photovoltaic
IP: Inverted pyramid
Si: Silicon
MACE: Metal-assisted chemical etching
PERC: Passivated emitter and rear cell
PERL: Passivated emitter and rear local-diffused solar cell
c-Si: Crystalline silicon
mc-Si: Multi-crystalline silicon
PECVD: Plasma-enhanced chemical vapor deposition
ALD: Atomic layer deposition
Si IP-strus: Silicon inverted pyramid structures
R_ave_: Averaged reflectance
EQE: external quantum efficiency
*τ*_eff_: The effective minority carrier lifetime
*Δn*: The injection level
FTIR: Fourier transform infrared spectroscopic
*N*_*f*_: Density of fixed charges
*N*_*it*_: Density of interface traps
*C-V*: Capacitance–voltage
IQE: Internal quantum efficiency
*V*_oc_: Open-circuit voltage
*I*_sc_: Short-circuit current
FF: Fill factor
*R*_*s*_: Series resistance
